# Prevalence of Antibiotic Resistance Genes and Bacterial Community Composition in a River Influenced by a Wastewater Treatment Plant 

**DOI:** 10.1371/journal.pone.0078906

**Published:** 2013-10-25

**Authors:** Elisabet Marti, Juan Jofre, Jose Luis Balcazar

**Affiliations:** 1 Catalan Institute for Water Research (ICRA), Scientific and Technological Park of the University of Girona, Girona, Spain; 2 Department of Microbiology, University of Barcelona, Barcelona, Spain; Oak Ridge National Laboratory, United States of America

## Abstract

Antibiotic resistance represents a global health problem, requiring better understanding of the ecology of antibiotic resistance genes (ARGs), their selection and their spread in the environment. Antibiotics are constantly released to the environment through wastewater treatment plant (WWTP) effluents. We investigated, therefore, the effect of these discharges on the prevalence of ARGs and bacterial community composition in biofilm and sediment samples of a receiving river. We used culture-independent approaches such as quantitative PCR to determine the prevalence of eleven ARGs and 16S rRNA gene-based pyrosequencing to examine the composition of bacterial communities. Concentration of antibiotics in WWTP influent and effluent were also determined. ARGs such as *qnrS, bla*_TEM_, *bla*_CTX-M_, *bla*_SHV_, *erm*(B), *sul*(I), *sul*(II), *tet*(O) and *tet*(W) were detected in all biofilm and sediment samples analyzed. Moreover, we observed a significant increase in the relative abundance of ARGs in biofilm samples collected downstream of the WWTP discharge. We also found significant differences with respect to community structure and composition between upstream and downstream samples. Therefore, our results indicate that WWTP discharges may contribute to the spread of ARGs into the environment and may also impact on the bacterial communities of the receiving river.

## Introduction

Antibiotic resistance represents a significant global health problem due to the use and misuse of antibiotics, which favors the emergence and spread of resistant bacteria. Since the first warning of antibiotic resistance [1], this phenomenon has increased dramatically and as a result, 70% of all hospital-acquired infections in the United States are resistant to at least one family of antibiotics [2]. The treatment of these infections leads to higher healthcare costs because these therapies require longer hospital stays and more expensive drugs. To confront this increasing problem, it is necessary to understand the ecology of antibiotic resistance, including their origins, evolution, selection and dissemination [3]. 

Although antibiotic resistance has involved extensive research in clinically relevant human pathogens, environmental reservoirs of antibiotic resistance determinants and their contribution to resistance in clinical settings have only been considered in the last decade [4–6]. It has been shown that antibiotic resistance genes (ARGs) have environmental origins but the introduction and accumulation of antimicrobials in the environment facilitates their spread [7]. As a consequence, ARGs can be found in almost all environments and they are currently considered as emerging pollutants [8,9]. Therefore, identifying sources of resistance genes, their environmental distribution and how anthropogenic inputs affect their spread will aid in establishing strategies to combat antibiotic resistance.

The location of ARGs on genetic elements that can be mobilized, such as transposons, integrons and plasmids, facilitates the transfer of resistance to other organisms of the same or different species [10]. Although antibiotic resistance studies have been focused on cultivable bacteria and/or indicator organisms in treated wastewater, the vast majority of environmental bacteria cannot be cultured under standard laboratory conditions. As a result, there is little information about how the discharge of wastewater effluents can affect bacterial communities and impact the prevalence of resistance genes in the environment.

It is well known that WWTPs reduce the total number of bacteria, especially coliforms, in their final effluent [11]. However, the treatment is not efficient enough to remove ARGs that are released to the receiving river [12,13]. In addition, WWTPs link human activities and the environment and may facilitate horizontal transfer of resistance determinants among a rich diversity of commensals, environmental microorganisms and clinically relevant pathogens [14]. In this regard, WWTP may contribute to the occurrence, spread and persistence of both antibiotic-resistant bacteria and antibiotic resistance determinants in the environment.

We used culture-independent approaches to determine the prevalence of ARGs and to examine how bacterial communities from biofilms and sediments respond to the discharge of WWTP effluents in the receiving river. ARGs and bacterial community composition in the upstream river were also analyzed to determine the contribution of wastewater discharge to antibiotic resistance in the downstream river samples. Biofilm and sediment were selected rather than water because they are substrates with a high bacterial density where the frequency of physical contacts between bacteria increase the possibility for horizontal gene transfer [15]. Actually, some studies have proposed aquatic biofilms as long-term reservoirs for ARGs in environment [9,16].

## Materials and Methods

### Study site and sampling

The study was carried out in the Ter River upstream and downstream of the Ripoll WWTP discharge. This river is the water supply for most cities in Catalonia. The WWTP has a primary and secondary treatment operating with conventional activated sludge and was planned for a population of 45,000 inhabitants. It receives an average daily flow about 8000 m^3^ made up primarily of domestic wastewater and a small amount of industrial and hospital wastewater, which are not pre-treated. Samples were obtained in June 2010, the end of the spring season, when water flow is maximum. Biofilm and sediment samples were collected in duplicate at the WWTP discharge point and at 100 m upstream and downstream of the WWTP. Both sample types were collected manually, scraping the surface of submerged stones for epilithic biofilm and collecting the top layer (0-5 cm) of sediment. Water samples for antibiotics quantification were taken from influent and effluent of the WWTP. All samples were stored on ice and transported to the laboratory for immediate processing.

### Ethics statement

Permission for the WWTP samples was granted by the Ripoll Treatment Plant (Girona, Spain), specifically Angel Maderiano de Pastor (Supervisor, Wastewater Treatment Division). No specific permits or permissions were required for the samples collected in the Ter River.

### Antibiotics quantification

To determine the efficiency of WWTP on antibiotics removal, some of these substances were quantified in WWTP influent and effluent. Quantification of antibiotics was carried out following the analytical methodology previously described [17]. Briefly, water samples were filtered through 1 μm glass microﬁbre ﬁlters followed by 0.45-μm nylon membrane filters (Whatman Maidstone, UK). Target compounds were extracted by solid-phase extraction using Oasis HLB cartridges (60mg, 3ml; Waters, Milford, MA, USA). Cartridges were loaded with 200 mL of water samples and a Baker vacuum system (J.T. Baker, Deventer, The Netherlands) was used to preconcentrate samples. The extracts were evaporated under a gentle nitrogen stream and reconstituted with 1 mL of methanol/water (25:75, v/v). Extracts were then analyzed by high-performance liquid chromatography tandem mass spectrometry using an Agilent HP 1100 HPLC (Agilent Technologies, Palo Alto, CA, USA) connected with a QTRAP hybrid triple quadrupole-linear ion trap mass spectrometer (Applied Biosystems/ MDS SCIEX, Foster City, CA, USA).

### DNA extraction

Biofilm and sediment samples were homogenized in phosphate-buffered saline solution (PBS; 10 mM sodium phosphate, 150 mM sodium chloride, pH 7.2) and the supernatants were then resuspended in lysis buffer (20 mM Tris-Cl, pH 8.0; 2 mM sodium EDTA; 1.2 % Triton X-100; and 20 mg/ml lysozyme). Genomic DNA was extracted using a standard phenol-chloroform method and the final concentration and purity were determined using a NanoDrop spectrophotometer (Thermo Scientific, Wilmington, DE, USA). DNA was extracted in duplicate from each independent sample, obtaining 4 analytical replicates, and all DNA samples were stored at -20 °C until further analysis. 

### Quantification of antibiotic resistance genes

Quantitative PCR was used to quantify eleven genes encoding resistance to the main antibiotic families used for treating human and animal infections such as beta-lactams (*bla*_TEM_, *bla*_CTX-M_ and *bla*_SHV_), fluoroquinolones (*qnrA*, *qnrB* and *qnrS*), tetracyclines [*tet*(O) and *tet*(W)], sulfonamides [*sul*(I) and *sul*(II)] and macrolides [*erm*(B)]. The 16S ribosomal RNA (rRNA) gene was also analyzed to quantify the total bacterial load and to normalize the abundance of ARGs in the collected samples. All qPCR assays were performed on an Mx3005P system (Agilent Technologies) using SYBR Green detection chemistry. Each reaction was carried out in a total volume of 30 µl, containing 1 µl of template, the corresponding concentration of each primer (from 0.2 to 0.6 µM) and 2× Brilliant III Ultra Fast QCPR Master Mix (Stratagene, La Jolla, CA, USA), except for the *bla*_TEM_ gene, which was amplified using the SYBR® Green Master Mix (Applied Biosystems). Primers and thermal cycling conditions for each gene are given in supplementary material, Table S1. In all cases, DNA extracted from samples was diluted 10- and 100-fold and positive controls were spiked with our DNA samples in order to screen for PCR inhibition by environmental matrices. Moreover, after the PCR a dissociation curve was constructed in the range of 60°C to 95°C to verify the specificity of the amplified products. 

Standard curves were generated using known quantities of cloned target genes. Briefly, amplicons from positive controls were ligated into pCR2.1-TOPO cloning vectors (Invitrogen, Carlsbad, CA, USA) and transformed into *Escherichia coli* competent cells following the manufacturer’s protocol (Invitrogen). Plasmids were extracted using the PureLink Plasmid kit (Invitrogen), and the concentration was determined using a Nanodrop spectrophotometer (Thermo Scientific). Copy number was then calculated as described previously [18]. Tenfold serial dilutions of plasmid DNA were amplified over seven orders of magnitude and in triplicate to generate a standard curve for each qPCR assay.

Mean values (copy number of each ARG) of four analytical replicates for each sample were compared using the one-way analysis of variance (ANOVA) or Kruskal-Wallis test as appropriate, because most data were not normally distributed. Data were analyzed using SPSS for Windows version 17.0 (SPSS, Chicago, IL, USA).

### Sequence analysis and phylogenetic classification

DNA extraction replicates from each sample were pooled and submitted to the Research and Testing Laboratory (Lubbock, TX, USA) for tag-pyrosequencing. Samples were amplified with primers 27F (3’-GAG TTT GAT CNT GGC TCAG-5’) and 519R (3’-GTN TTA CNG CGG CKG CTG-5’), and the amplicons were sequenced using Roche 454 GS-FLX Titanium technology [19]. Sequences were quality trimmed using the MOTHUR software package [20]. Briefly, we removed sequences that did not perfectly match the PCR primer at the beginning of a read, sequences that contained more than one ambiguous base, sequences having a homopolymer stretch longer than 8 bp, and sequences with an average quality score below 30. We also included only the first 250 bp after the proximal PCR primer, because the quality of sequences degrades beyond this point. Then, sequences were aligned using the SILVA reference database [21] and potential chimeric sequences were detected and removed by using chimera.uchime incorporated into MOTHUR. Qualified sequences were assigned to operational taxonomic units (OTUs) based on a 97% sequence similarity. The Shannon diversity index (H’) and the Chao1 richness estimator were also calculated. The Bray-Curtis distance, which incorporates both membership and abundance, was used to compare beta diversity among samples. The parsimony test, as implemented by MOTHUR, was used to assess whether two or more communities have the same structure. A Bonferroni correction was applied to adjust the significance level for multiple pairwise comparisons (*p*≤0.05/15 [0.0033]). The Ribosomal Database Project (RDP) pipeline and Classifier function were used to align and assign identities at a confidence threshold of 50% [22]. The sequences from this study have been deposited in the NCBI Short Read Archive under accession number SRA067245.

## Results

### Antibiotic concentrations

Antibiotic compounds, such as clarithromycin, sulfamethoxazole, trimethroprim, metronidazole and ciprofloxacin were detected in WWTP influent and effluent samples at concentrations ranging from 20.8 to 913 ng/L (Table 1). Although concentrations found in the WWTP influent were higher than those in the effluent, relatively high levels of antibiotic compounds were detected in treated water from the WWTP.

**Table 1:** Concentrations of antibiotics determined in WWTP influent and effluent water samples.

Antibiotic	Influent (ng/L)	Effluent (ng/L)
Clarithromycin	181.9	166.0
Trimethoprim	22.0	20.8
Metronidazole	161.0	43.3
Sulfamethoxazole	136.0	57.8
Ciprofloxacin	913.0	231.0

### Quantification of ARGs

**Figure 1:**
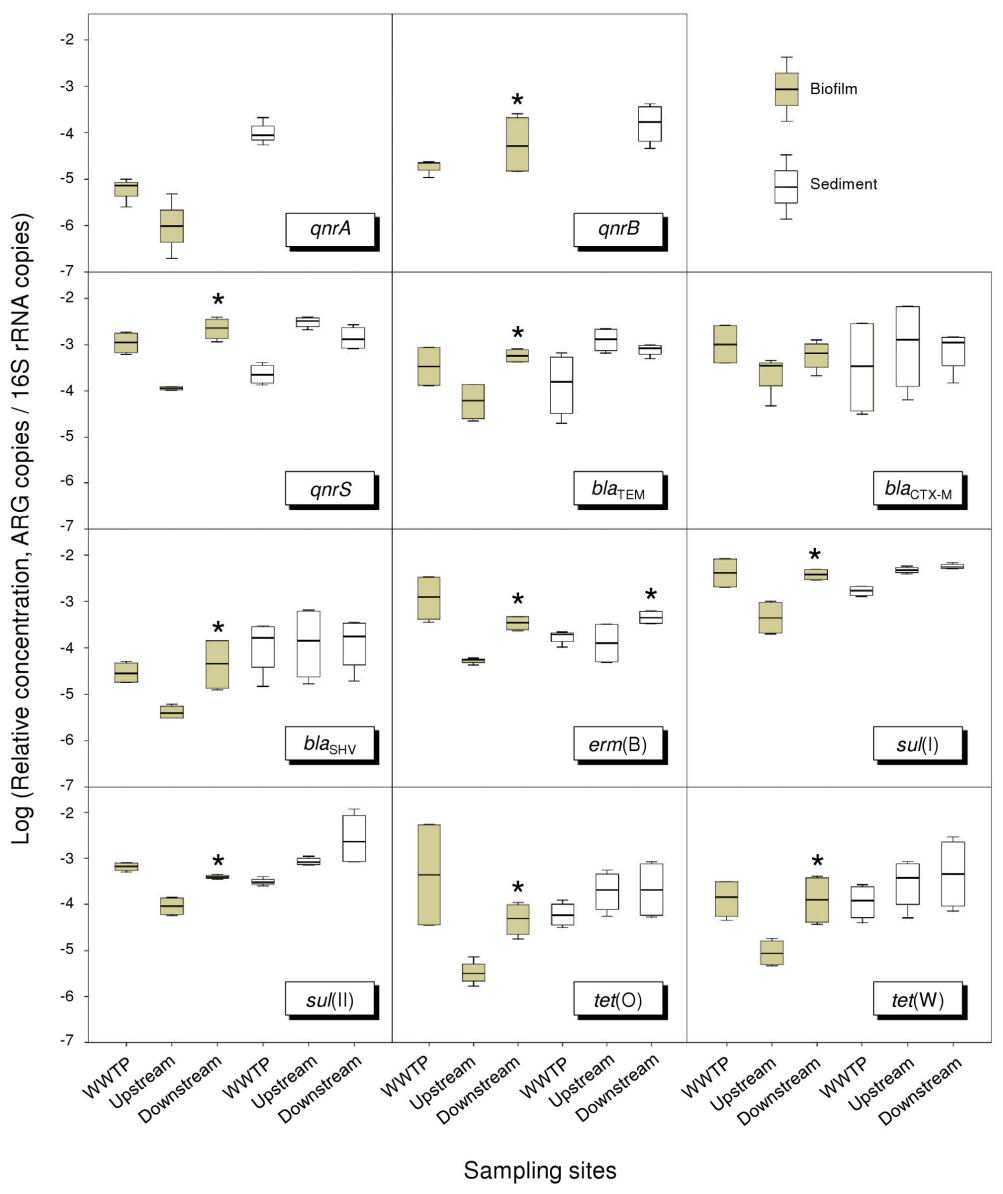
Relative concentration of ARGs in biofilm and sediment samples. Within the box plot chart, the crosspieces of each box plot represent (from top to bottom) maximum, upper-quartile, median (black bar), lower-quartile, and minimum values. An asterisk (*) denotes a statistically significant difference between upstream and downstream sites (*P*<0.05).

ARGs, including *qnrA*, *qnrB*, *qnrS, bla*_TEM_, *bla*_SHV_, *bla*_CTX-M_, *erm*(B), *sul*(I), *sul*(II), *tet*(O) and *tet*(W), and the 16S rRNA gene were quantified by qPCR in the biofilm and sediment samples. High *R* ^2^ values (average 0.995) and high efficiencies (from 95 to 103%) were obtained over at least 5 orders of magnitude in all qPCR assays, indicating the validity of these quantifications (data not shown). Results revealed that the total copy numbers of bacterial 16S rRNA genes were consistent in all samples and ranged from 1.45×10^9^ to 1.21×10^11^ copy numbers per gram. Relative concentrations of ARGs (normalized to the 16S rRNA gene copy number) are shown in Figure 1. From this figure it can be seen that, except *qnrA* and *qnrB*, all ARGs were detected in the samples analyzed. It is noteworthy that relative abundances of the *qnrB*, *qnrS, bla*_TEM_, *bla*_SHV_, *erm*(B), *sul*(I), *sul*(II), *tet*(O) and *tet*(W) genes were significantly higher (*p*<0.05) in the downstream biofilm samples than those found in the upstream samples. Regarding sediment samples, no significant differences in relative concentrations of ARGs were observed among them, except for the *erm*(B) gene.

### Bacterial community composition

A total of 77,056 reads from biofilm and sediment samples were obtained after quality trimming and filtering the initial reads. The library size of each sample was then normalized to the smallest number of sequences obtained from biofilm and sediment samples (4,328 and 7,587 sequences, respectively) in order to minimize any bias due to the difference in the total number of sequences. The number of OTUs observed at a 97% taxonomic cutoff ranged from 262 (in the upstream biofilm) to 2,527 (in sediment from the WWTP discharge point). Shannon diversity index and Chao richness estimators were also determined (Table 2), demonstrating that the sediment samples had a higher bacterial diversity and richness than the biofilm samples. 

**Table 2:** Measures of α diversity for the biofilm and sediment samples.

	Biofilm				Sediment		
	WWTP	Upstream	Downstream		WWTP	Upstream	Downstream
No. of sequences	4328	4328	4328		7587	7587	7587
OTUs	560	262	740		2527	1795	2202
H’	4.75	1.53	3.97		7.06	5.81	6.70
Chao1	1145	852	1988		6372	4378	5649

**Figure 2:**
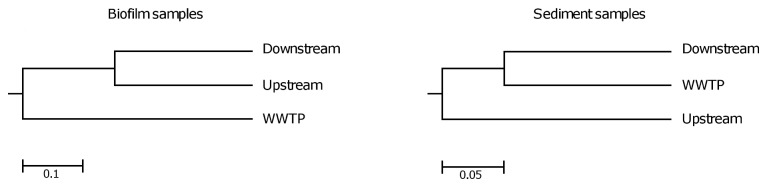
The dendrograms represent the similarity among the samples based on the Bray-Curtis coefficient. Scale bars indicate the similarity obtained from calculated matrices.

To compare the bacterial community structure and determine the effect of WWTP discharges into the environment, we used the phylogeny-based parsimony test which showed a significant difference (*p*<0.001) in community structure between all analyzed samples. When the pairwise distances for all samples were calculated using the Bray-Curtis distance metric, the results revealed the impact of the WWTP effluents on the bacterial community structure in the sediment of receiving river, as visualized by the terminal branch lengths (Figure 2). 

**Figure 3:**
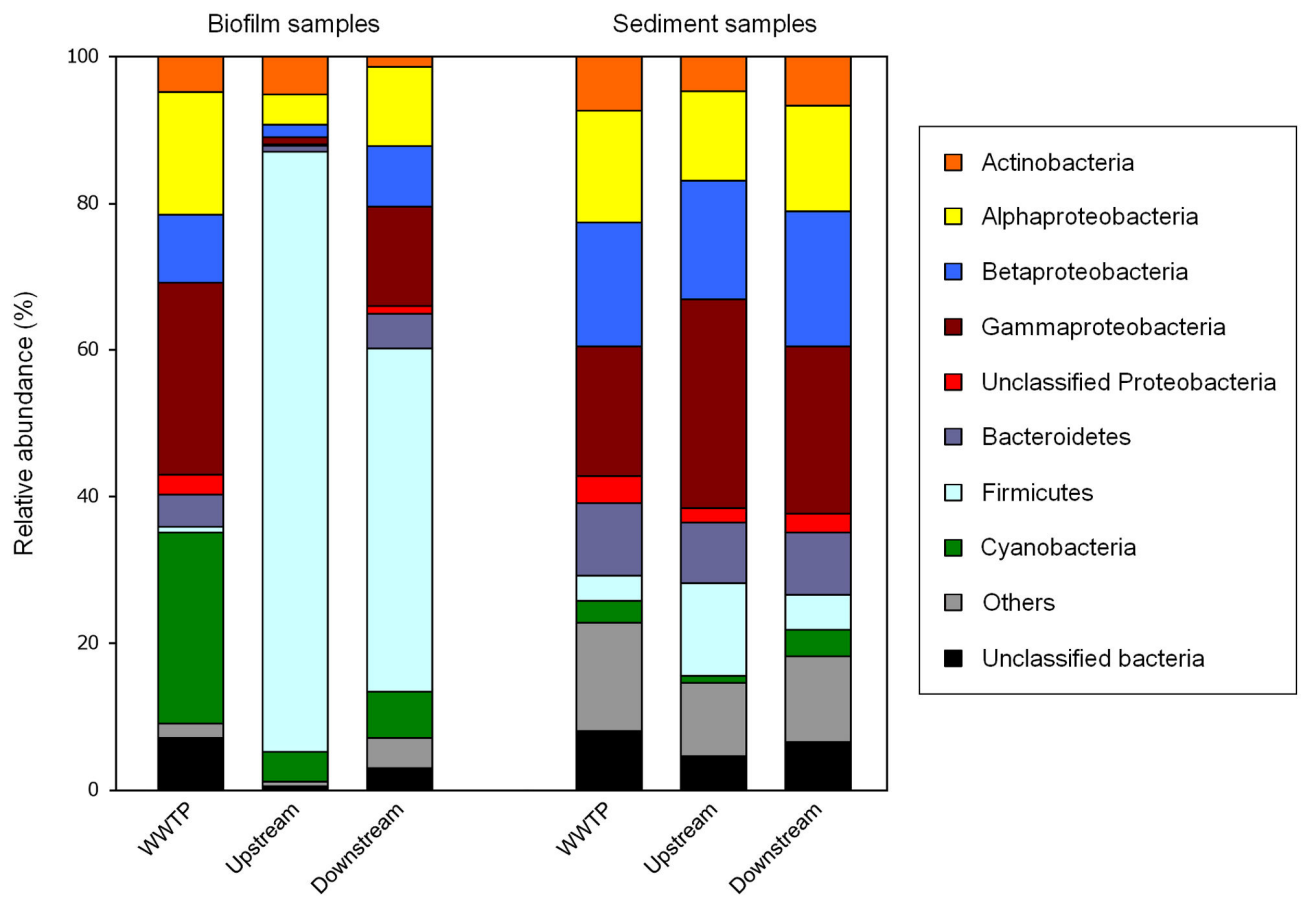
Relative abundance of major bacterial lineages (phyla; and classes for Proteobacteria) found in the biofilm and sediment samples.

Phylogenetic classification of sequences was determined using the RDP Classifier tool with a bootstrap cutoff of 50% (Figure 3). Overall taxonomic characterization of the bacterial community was conducted at the phylum level, and only Proteobacteria were classiﬁed at the class or order level. Biofilm sequences showed great differences between WWTP and river samples. Although the biofilm from the WWTP discharge point was dominated by *Cyanobacteria* and Proteobacteria, the most abundant groups in both upstream and downstream biofilms were Firmicutes. Gammaproteobacteria were mainly represented by the genera *Aeromonas* (16%) and *Acinetobacter* (8%) in the biofilm from the WWTP discharge point, whereas *Exiguobacterium* was the most predominant genus (data not shown), accounting for 85% of the observed OTUs in upstream samples and 46% in downstream samples. On the other hand, members of the Proteobacteria and Actinobacteria phyla were abundant in all sediment samples. Sequences from sediment samples were dominated by common genera such as *Aeromonas*, *Exiguobacterium*, *Piscinibacter*, *Pseudohodoferax*, *Acinetobacter* and *Pseudomonas*.

## Discussion

In this study we investigated the prevalence of eleven ARGs and the bacterial community composition in biofilm and sediment samples from a river influenced by a WWTP. It has been some years since Iwane and colleagues [23] showed that the increase of antibiotic resistant bacteria in the Tama River was associated with WWTP effluent discharges. Since then, a wide range of genetic methods have been developed and some culture-independent studies have been performed to explore the impact of antibiotics in the environment; however, most of these studies have been limited to a few ARGs [24–26].

Although WWTPs efficiently reduce high nutrient concentrations from raw sewage, our study demonstrates, to a certain extent, that antibiotics are not completely degraded during wastewater treatment. Previous studies have shown that antibiotic levels in treated wastewater are typically in the nanogram per liter range [15] and WWTP effluents are the major pathway for pharmaceuticals to reach the aquatic environment [27,28]. Effluents are diluted once they reach the river, and even though antibiotic concentrations in the environment are low, in general below the minimum inhibitory concentration for most sensitive bacteria, they may still exert a selection pressure [29,30] and impact on the microbial community [31].

Regarding ARG concentrations, we identified nine of the eleven ARGs studied in all samples analyzed. Detection of these genes in upstream biofilm and sediment samples supports the idea of an existing background level of antibiotic resistance naturally occurring in the environment [10]. However, other anthropogenic activities in the river upstream, such as livestock rearing, may have perturbed the bacterial community. We also report that there was a significant increase in the relative concentration for almost all ARGs studied in the biofilm samples after the WWTP effluent discharges. This is consistent with the observations of Engemann et al. [9] which suggested the migration of ARGs from the water column to biofilms. Our findings thus, together with those previously reported, suggest that biofilms could be considered as good indicators of antibiotic resistance acquisition. Moreover, our results reinforce the view that environmental compartments directly impacted by anthropogenic activities, such as wastewater discharges, show a higher concentration of ARGs [8,24,32]. 

Previous studies have also suggested that aquatic sediments could be important reservoirs for ARGs due to their ability to retain antibiotic compounds as well as representing an important environmental matrix within which horizontal gene transfer can occur [33]. LaPara et al. [32] detected ARGs encoding resistance to tetracyclines in water and sediments samples in locations influenced by WWTP effluents. Although ARGs were found in most of the sediment samples analyzed, we could not determine the effect of WWTP in the receiving river as some ARG levels in the discharge point had similar values in terms of relative concentration to those found in the upstream and downstream sediment samples. 

We also investigated the potential effect of WWTP effluents on bacterial communities associated with the receiving river by using 454-pyrosequencing technology. Based on the Chao1 and Shannon indices, the bacterial communities in the sediment samples had higher richness and diversity than those in the biofilm samples. This could be because, although biofilms can be composed of multiple microbial species, they may also be dominated by a few genera in the primary colonization phase [34].

It has also been reported that sediments from aquatic environments have a complex and dynamic community of microorganisms [35]. In this study, the bacterial communities in the biofilm and sediment samples from the WWTP discharge point and downstream river had a higher diversity than those in the upstream samples, suggesting that WWTP effluent discharges may have promoted bacterial growth by supplying nutrients [36].

Despite the high degree of similarity among the bacterial communities from river and WWTP sediment samples at the phylum level, differences with respect to community structure and composition were significant at the genus level (97% similarity) as revealed by the phylogeny-based parsimony test. Similarly, Kristiansson et al. [6] found differences in the distribution of bacterial genera between the upstream and downstream sediments from an Indian river influenced by WWTP effluents. Moreover, our results also agreed with the observation of Kristiansson et al. [6] that all sediment communities were dominated by Proteobacteria, Bacteroidetes and Firmicutes. With regard to the biofilm samples, differences in community structures were evident even at the phylum level. *Cyanobacteria* and Proteobacteria were dominant in the WWTP discharge point, whereas Firmicutes was the dominant group in both upstream and downstream samples. A high representation of *Cyanobacteria* was found in biofilm samples from the WWTP, which may be a consequence of light intensity, as the WWTP effluent canal is less deep than the river, promoting the growth of phototrophic microorganisms. Concerning the relationship between changes in ARG concentrations and bacterial community composition, we found a higher proportion of Gammaproteobacteria in downstream biofilm samples compared with upstream samples, which could explain the increase of ARGs as several members of this class harbour ARGs [37,38]. A computer search using the Antibiotic Resistance Genes Database [39] also revealed that most of the bacterial species harboring ARGs belong to this class. In this sense, the similarity among the proportion of Gammaproteobacteria in bacterial communities from sediment samples could be related with the similar values of ARGs found in the different points analysed. 

Additionally, we could detect and estimate the number of bacterial genera in each sample, which have been previously identified as harbouring ARGs. The genus *Exiguobacterium*, which was the dominant OTU in biofilm river samples and also appeared in sediment samples, has been recently characterized as a carrier of some ARGs encoding resistance to beta-lactams and sulphonamides [40]. The genus *Aeromonas*, which was detected in all samples, has been widely studied because most of ARGs we analyzed have been detected in several species of this genus [41,42]. Moreover, members of the genus *Acinetobacter*, which were present in high percentages in biofilm and sediment samples, have been also described as multidrug-resistant microorganisms encoding resistance to beta-lactams, aminoglycosides, ﬂuoroquinolones and carbapenems [43]. Given this, microorganisms belonging to these genera may have contributed to the occurrence, spread and persistence of ARGs.

In conclusion, eleven different ARGs encoding resistance to the most important antibiotic families were analyzed using a culture-independent method, which contributes to a better understanding on the spread of antibiotic resistance in the environment. Of special concern is that our findings, together with reports from other settings, demonstrate that WWTP discharges may increase the prevalence of ARGs and bacterial community composition of the receiving river. However, further research is needed to evaluate if the increase of ARGs in aquatic ecosystems is due to the release of resistant bacteria from WWTP or due to antibiotics discharged in their effluents promoting horizontal gene transfer once they reach the river. 

## Supporting Information

Table S1 **Primer sequences and qPCR conditions used in this study.**
(DOC)

## References

[1] McCoy E (1954) Changes in the host flora induced by chemotherapeutic agents. Annu Rev Microbiol 8: 257–272. doi:10.1146/annurev.mi.08.100154.001353. PubMed: 13198107. 13198107

[2] Leeb M (2004) Antibiotics: a shot in the arm. Nature 431: 892–893. doi:10.1038/431892a. PubMed: 15496888. 15496888

[3] Aminov RI, Mackie RI (2007) Evolution and ecology of antibiotic resistance genes. FEMS Microbiol Lett 271: 147–161. doi:10.1111/j.1574-6968.2007.00757.x. PubMed: 17490428. 17490428

[4] Huerta B, Marti E, Gros M, López P, Pompêo M et al. (2013) Exploring the links between antibiotic occurrence, antibiotic resistance, and bacterial communities in water supply reservoirs. Sci Total Environ 456-457: 161–170. doi:10.1016/j.scitotenv.2013.03.071. PubMed: 23591067. 23591067

[5] Di Cesare A, Luna GM, Vignaroli C, Pasquaroli S, Tota S et al. (2013) Aquaculture can promote the presence and spread of antibiotic-resistant enterococci in marine sediments. PLOS ONE 8: e62838. doi:10.1371/journal.pone.0062838. PubMed: 23638152. 23638152 PMC3637307

[6] Kristiansson E, Fick J, Janzon A, Grabic R, Rutgersson C et al. (2011) Pyrosequencing of antibiotic-contaminated river sediments reveals high levels of resistance and gene transfer elements. PLOS ONE 6: e17038. doi:10.1371/journal.pone.0017038. PubMed: 21359229. 21359229 PMC3040208

[7] Baquero F, Martínez JL, Cantón R (2008) Antibiotics and antibiotic resistance in water environments. Curr Opin Biotechnol 19: 260–265. doi:10.1016/j.copbio.2008.05.006. PubMed: 18534838. 18534838

[8] Pruden A, Pei R, Storteboom H, Carlson KH (2006) Antibiotic resistance genes as emerging contaminants: studies in northern Colorado. Environ Sci Technol 40: 7445–7450. doi:10.1021/es060413l. PubMed: 17181002. 17181002

[9] Engemann CA, Keen PL, Knapp CW, Hall KJ, Graham DW (2008) Fate of tetracycline resistance genes in aquatic systems: Migration from the water column to peripheral biofilms. Environ Sci Technol 42: 5131–5136. doi:10.1021/es800238e. PubMed: 18754359. 18754359

[10] Allen HK, Donato J, Wang HH, Cloud-Hansen KA, Davies J et al. (2010) Call of the wild: antibiotic resistance genes in natural environments. Nat Rev Microbiol 8: 251–259. doi:10.1038/nrmicro2312. PubMed: 20190823. 20190823

[11] Zhang K, Farahbakhsh K (2007) Removal of native coliphages and coliform bacteria from municipal wastewater by various wastewater treatment processes: implications to water reuse. Water Res 41: 2816–2824. doi:10.1016/j.watres.2007.03.010. PubMed: 17449083. 17449083

[12] Munir M, Wong K, Xagoraraki I (2011) Release of antibiotic resistant bacteria and genes in the effluent and biosolids of five wastewater utilities in Michigan. Water Res 45: 681–693. doi:10.1016/j.watres.2010.08.033. PubMed: 20850863. 20850863

[13] Mokracka J, Koczura R, Kaznowski A (2012) Multiresistant *Enterobacteriaceae* with class 1 and class 2 integrons in a municipal wastewater treatment plant. Water Res 46: 3353–3363. doi:10.1016/j.watres.2012.03.037. PubMed: 22507248. 22507248

[14] LaPara T, Burch T (2012) Municipal wastewater as a reservoir of antibiotic resistance. In: Keen PL Montforts M Antimicrobial Resistance in the environment. Hoboken, NJ: Wiley-Blackwell pp. 241–250.

[15] Janzon A, Kristiansson E, Larsson DGJ (2012) Environmental microbial communities living under very high antibiotic selection pressure. In: Keen PL Montforts M Antimicrobial Resistance in the environment. Hoboken, NJ: Wiley-Blackwell pp. 483–501.

[16] Zhang XX, Zhang T, Fang HH (2009) Antibiotic resistance genes in water environment. Appl Microbiol Biotechnol 82: 397–414. doi:10.1007/s00253-008-1829-z. PubMed: 19130050. 19130050

[17] Gros M, Petrović M, Barceló D (2009) Tracing pharmaceutical residues of different therapeutic classes in environmental waters by using liquid chromatography/quadrupole-linear ion trap mass spectrometry and automated library searching. Anal Chem 81: 898–912. doi:10.1021/ac801358e. PubMed: 19113952. 19113952

[18] Marti E, Balcázar JL (2013) Real-time PCR assays for quantification of *qnr* genes in environmental water samples and chicken feces. Appl Environ Microbiol 79: 1743–1745. doi:10.1128/AEM.03409-12. PubMed: 23275512. 23275512 PMC3591933

[19] Acosta-Martinez V, Dowd S, Sun Y, Allen V (2008) Tag-encoded pyrosequencing analysis of bacterial diversity in a single soil type as affected by management and land use. Soil Biol Biochem 40: 2762–2770. doi:10.1016/j.soilbio.2008.07.022.

[20] Schloss PD, Westcott SL, Ryabin T, Hall JR, Hartmann M et al. (2009) Introducing mothur: open-source, platform-independent, community-supported software for describing and comparing microbial communities. Appl Environ Microbiol 75: 7537–7541. doi:10.1128/AEM.01541-09. PubMed: 19801464. 19801464 PMC2786419

[21] Pruesse E, Quast C, Knittel K, Fuchs BM, Ludwig W et al. (2007) SILVA: a comprehensive online resource for quality checked and aligned ribosomal RNA sequence data compatible with ARB. Nucleic Acids Res 35: 7188–7196. doi:10.1093/nar/gkm864. PubMed: 17947321. 17947321 PMC2175337

[22] Wang Q, Garrity GM, Tiedje JM, Cole JR (2007) Naive Bayesian classifier for rapid assignment of rRNA sequences into the new bacterial taxonomy. Appl Environ Microbiol 73: 5261–5267. doi:10.1128/AEM.00062-07. PubMed: 17586664. 17586664 PMC1950982

[23] Iwane T, Urase T, Yamamoto K (2001) Possible impact of treated wastewater discharge on incidence of antibiotic resistant bacteria in river water. Water Sci Technol 43: 91–99. PubMed: 11380211. 11380211

[24] Pei R, Kim SC, Carlson KH, Pruden A (2006) Effect of river landscape on the sediment concentrations of antibiotics and corresponding antibiotic resistance genes (ARG). Water Res 40: 2427–2435. doi:10.1016/j.watres.2006.04.017. PubMed: 16753197. 16753197

[25] Chen J, Yu Z, Michel FC, Wittum T, Morrison M (2007) Development and application of real-time PCR assays for quantification of *erm* genes conferring resistance to macrolide-lincosamides-streptogramin B in livestock manure and manure management systems. Appl Environ Microbiol 14: 4407–4416. 10.1128/AEM.02799-06 PMC1932836 17496134

[26] Li J, Wang T, Shao B, Shen J, Wang S et al. (2012) Plasmid-mediated quinolone resistance genes and antibiotic residues in wastewater and soil adjacent to Swine feedlots: Potential transfer to agricultural lands. Environ Health Perspect 120: 1144–1149. doi:10.1289/ehp.1104776. PubMed: 22569244. 22569244 PMC3440090

[27] Petrovic M, Gonzalez S, Barceló D (2003) Analysis and removal of emerging contaminants in wastewater and drinking water. Trends Anal Chem 22: 685–696. doi:10.1016/S0165-9936(03)01105-1.

[28] Gros M, Rodríguez-Mozaz S, Barceló D (2012) Fast and comprehensive multi-residue analysis of a broad range of human and veterinary pharmaceuticals and some of their metabolites in surface and treated waters by ultra-high-performance liquid chromatography coupled to quadrupole-linear ion trap tandem mass spectrometry. J Chromatogr A 1248: 104–121. doi:10.1016/j.chroma.2012.05.084. PubMed: 22704668. 22704668

[29] Martínez JL (2008) Antibiotics and antibiotic resistance genes in natural environments. Science 321: 365–367. doi:10.1126/science.1159483. PubMed: 18635792. 18635792

[30] Kümmerer K (2009) Antibiotics in the aquatic environment - A review - Part I. Chemosphere 75: 417–434. doi:10.1016/j.chemosphere.2008.11.086. PubMed: 19185900. 19185900

[31] Davies J, Spiegelman GB, Yim G (2006) The world of subinhibitory antibiotic concentrations. Curr Opin Microbiol 9: 445–453. doi:10.1016/j.mib.2006.08.006. PubMed: 16942902. 16942902

[32] LaPara TM, Burch TR, McNamara PJ, Tan DT, Yan M et al. (2011) Tertiary-treated municipal wastewater is a significant point source of antibiotic resistance genes into Duluth-Superior Harbor. Environ Sci Technol 45: 9543–9549. doi:10.1021/es202775r. PubMed: 21981654. 21981654

[33] Taylor NG, Verner-Jeffreys DW, Baker-Austin C (2011) Aquatic systems: maintaining, mixing and mobilising antimicrobial resistance? Trends Ecol Evol 26: 278–284. doi:10.1016/j.tree.2011.03.004. PubMed: 21458879. 21458879

[34] Sigee DC (2005) Freshwater Microbiology: Biodiversity and Dynamic Interactions of Microorganisms in the Aquatic Environment. Chichester, England: John Wiley & Sons Ltd. p. 524.

[35] Lu SY, Zhang YL, Geng SN, Li TY, Ye ZM et al. (2010) High diversity of extended-spectrum beta-lactamase-producing bacteria in an urban river sediment habitat. Appl Environ Microbiol 76: 5972–5976. doi:10.1128/AEM.00711-10. PubMed: 20639374. 20639374 PMC2935080

[36] Wakelin SA, Colloff MJ, Kookana RS (2008) Effect of wastewater treatment plant effluent on microbial function and community structure in the sediment of a freshwater stream with variable seasonal flow. Appl Environ Microbiol 74: 2659–2668. doi:10.1128/AEM.02348-07. PubMed: 18344343. 18344343 PMC2394891

[37] Li D, Yu T, Zhang Y, Yang M, Li Z, Liu M, Qi R (2010) Antibiotic resistance characteristics of environmental bacteria from an oxytetracycline production wastewater treatment plant and the receiving river. Appl Environ Microbiol 76: 3444–3451. doi:10.1128/AEM.02964-09. PubMed: 20400569. 20400569 PMC2876458

[38] Tamminen M, Virta M, Fani R, Fondi M (2012) Large-scale analysis of plasmid relationships through gene-sharing networks. Mol Biol Evol 29: 1225–1240. doi:10.1093/molbev/msr292. PubMed: 22130968. 22130968

[39] Liu B, Pop M (2009) ARDB--Antibiotic Resistance Genes Database. Nucleic Acids Res 37: D443–D447. doi:10.1093/nar/gkn656. PubMed: 18832362. 18832362 PMC2686595

[40] Carneiro AR, Ramos RT, Dall'Agnol H, Pinto AC, de Castro Soares S et al. (2012) Genome sequence of *Exiguobacterium* *antarcticum* B7, isolated from a biofilm in Ginger Lake, King George Island, Antarctica. J Bacteriol 194: 6689–6690. doi:10.1128/JB.01791-12. PubMed: 23144424. 23144424 PMC3497522

[41] Cattoir V, Poirel L, Aubert C, Soussy CJ, Nordmann P (2008) Unexpected occurrence of plasmid-mediated quinolone resistance determinants in environmental *Aeromonas* spp. Emerg Infect Dis 14: 231–237. doi:10.3201/eid1402.070677. PubMed: 18258115. 18258115 PMC2600179

[42] Marti E, Balcázar JL (2012) Multidrug resistance-encoding plasmid from *Aeromonas* sp. strain P2G1. Clin Microbiol Infect 18: E366–E368. doi:10.1111/j.1469-0691.2012.03935.x. PubMed: 22725683. 22725683

[43] Mak JK, Kim MJ, Pham J, Tapsall J, White PA (2009) Antibiotic resistance determinants in nosocomial strains of multidrug-resistant *Acinetobacter* *baumannii* . J Antimicrob Chemother 63: 47–54. PubMed: 18988680. 18988680 10.1093/jac/dkn454

