# Supplementary material for: Prevalence of Antibiotic Resistance Genes and Bacterial Community Composition in a River Influenced by a Wastewater Treatment Plant
Source: PLoS One. 2013 Oct 25;8(10):e78906. doi: 10.1371/journal.pone.0078906 (PMC3808343; doi:10.1371/journal.pone.0078906)
Supplement: Table S1 — Primer sequences and qPCR conditions used in this study. (DOC) [file pone.0078906.s001.doc]

**Supplementary Table S1**. qPCR primer sequences, targets and conditions of reactions.

| **Target gene** | **Primers** | **Sequence** | **Conditions** | **Reference** |
| --- | --- | --- | --- | --- |
| 16S rRNA | F1048 | GTGSTGCAYGGYTGTCGTCA | 95ºC 3 min (1 cycle); 95ºC 15 s and 60ºC 1 min (35 cycles) | [1] |
|  | R1194 | ACGTCRTCCMCACCTTCCTC |
| *qnrA* | qnrAf-RT | ATTTCTCACGCCAGGATTTG | 95ºC 3 min (1 cycle); 95ºC 15 s and 64ºC 20 s (40 cycles) | Modified from Robicsek *et al*. [2] |
|  | qnrAr-RT | GCAGATCGGCATAGCTGAAG |
| *qnrB* | qnrBmF | GGMATHGAAATTCGCCACTG | 95ºC 3 min (1 cycle); 95ºC 15 s and 60ºC 20 s (40 cycles) | Modified from Cattoir *et al*. [3] |
|  | qnrBmR | TTYGCBGYYCGCCAGTCGAA |
| *qnrS* | qnrSf-RT | ATGCAAGTTTCCAACAATGC | 95ºC 3 min (1 cycle); 95ºC 15 s and 62ºC 20 s (40 cycles) | [4] |
|  | qnrSr-RT | CTATCCAGCGATTTTCAAACA |
| *bla*_TEM_ | bla-TEM, FX | GCKGCCAACTTACTTCTGACAACG | 95ºC 3 min (1 cycle); 95ºC 15 s and 60ºC 20 s (40 cycles) | [5] |
|  | bla-TEM, RX | CTTTATCCGCCTCCATCCAGTCTA |
| *bla*_CTX_ | RTCTXM-F | CTATGGCACCACCAACGATA | 95ºC 3 min (1 cycle); 95ºC 15 s and 60ºC 20 s (40 cycles) | Modified from Kim *et al*. [6] |
|  | RTCTXM-R | ACGGCTTTCTGCCTTAGGTT |
| *bla*_SHV_ | RTblaSHVF | CGCTTTCCCATGATGAGCACCTTT | 95ºC 3 min (1 cycle); 95ºC 15 s and 64ºC 30 s (40 cycles) | [5] |
|  | RTblaSHVR | TCCTGCTGGCGATAGTGGATCTTT |
| *sul*(I) | Sul(I)-FW | CGCACCGGAAACATCGCTGCAC | 95ºC 3 min (1 cycle); 95ºC 15 s and 65ºC 20 s (40 cycles) | [7] |
|  | Sul(I)-RV | TGAAGTTCCGCCGCAAGGCTCG |
| *sul*(II) | Sul(II)-FW | TCCGGTGGAGGCCGGTATCTGG | 95ºC 3 min (1 cycle); 95ºC 15 s and 58ºC 20 s (40 cycles) | [7] |
|  | Sul(II)-RV | CGGGAATGCCATCTGCCTTGAG |
| *tet*(O) | tet(O)-FW | ACGGARAGTTTATTGTATACC | 95ºC 3 min (1 cycle); 95ºC 15 s and 50ºC 20 s (40 cycles) | [8] |
|  | tet(O)-RV | TGGCGTATCTATAATGTTGAC |
| *tet*(W) | tet(W)-FW | GAGAGCCTGCTATATGCCAGC | 95ºC 3 min (1 cycle); 95ºC 15 s and 60ºC 20 s (40 cycles) | [8] |
|  | tet(W)-RV | GGGCGTATCCACAATGTTAAC |
| *erm*(B) | erm(B)-91f | GATACCGTTTACGAAATTGG | 95ºC 3 min (1 cycle); 95ºC 15 s and 58ºC 20 s (40 cycles) | [9] |
|  | erm(B)-454r | GAATCGAGACTTGAGTGTGC |

**References**

1. Maeda, H., Fujimoto, C., Haruki, Y., Maeda, T., Kokeguchi, S., Petelin, M., *et al.* (2003) Quantitative real-time PCR using TaqMan and SYBR Green for *Actinobacillus actinomycetemcomitans, Porphyromonas gingivalis, Prevotella intermedia, tetQ* gene and total bacteria. *FEMS Immunol. Med. Microbiol.* 39, 81-86.
2. Robicsek, A., Strahilevitz, J., Sahm, D. F., Jacoby, G.A., & Hooper, D.C. (2006) *qnr* Prevalence in Ceftazidime-Resistant *Enterobacteriaceae* Isolates from the United States. *Antimicrob. Agents Chemother.* 50, 2872-2874.
3. Cattoir, V., Poirel, L., Rotimi, V., Soussy, C.J., & Nordmann, P. (2007) Multiplex PCR for detection of plasmid-mediated quinolone resistance *qnr* genes in ESBL-producing enterobacterial isolates*. J. Antimicrob. Chemother.* 60, 394-397.
4. Marti, E., & Balcazar, J.L. (2013) Real-time PCR assays for quantification of *qnr* genes in environmental water samples and chicken feces. *Appl. Environ. Microbiol.* 79, 1743-1745.
5. Xi, C., Xi, C.W., Zhang, Y.L., Marrs, C.F., Ye, W., Simon, C., Foxman, B., & Nriagu, J. (2009) Prevalence of antibiotic resistance in drinking water treatment and distribution systems. *Appl. Environ. Microbiol.* 75, 5714-5718.
6. Kim, J., Lim, Y.M., Jeong, Y.S., & Seol, S.Y. (2005) Occurrence of CTX-M-3, CTX-M-15, CTX-M-14 and CTX-M-9 extended-spectrum b-lactamases in Enterobacteriaceae clinical isolates in Korea. *Antimicrob. Agents Chemother.* 49, 1572-1575.
7. Pei, R., Kim, S.C., Carlson, K.H., & Pruden, A. (2006) Effect of river landscape on the sediment concentrations of antibiotics and corresponding antibiotic resistance genes (ARG)*.* *Water Res.* 40, 2427-2435.
8. Aminov, R.I., Garrigues-Jeanjean N., & Mackie RI. (2001) Molecular ecology of tetracycline resistance: Development and validation of primers for detection of tetracycline resistance genes encoding ribosomal protection proteins. *Appl. Environ. Microbiol.* 67, 22-32.
9. Chen, J., Yu, Z., Michel, F.C., Wittum, T., & Morrison, M. (2007) Development and application of real-time PCR assays for quantification of *erm* genes conferring resistance to macrolide-lincosamides-streptogramin B in livestock manure and manure management systems. *Appl. Environ. Microbiol.* 14, 4407-4416.
